# *In vitro* Antifungal Activity of Olive (*Olea europaea*) Leaf Extracts Loaded in Chitosan Nanoparticles

**DOI:** 10.3389/fbioe.2020.00151

**Published:** 2020-03-03

**Authors:** Innocenzo Muzzalupo, Giuliana Badolati, Adriana Chiappetta, Nevio Picci, Rita Muzzalupo

**Affiliations:** ^1^Dipartimento di Farmacia, Scienze della Salute e della Nutrizione – Universitá della Calabria (DFSSN-UNICAL), Ed. Polifunzionale, Arcavacata di Rende (CS), Rende, Italy; ^2^Centro di Ricerca Olivicoltura, Frutticoltura, Agrumicoltura, Consiglio per la Ricerca in Agricoltura e L’analisi dell’Economia Agraria (CREA-OFA), Rende, Italy; ^3^Dipartimento di Biologia, Ecologia e Scienza della Terra, Università della Calabria, Arcavacata di Rende, Italy

**Keywords:** antifungal activity, olive leaf extracts, oleuropein, biofungicides, nanoformulates

## Abstract

Olive leaf extract is characterized by a high content of phenols and flavonoids (oleuropein, luteolin, and their derivatives). These compounds are defined as secondary metabolites and exert such as anti-inflammatory, antioxidant, and antimicrobial activities. We investigated the *in vitro* antifungal activity of two olive leaf extracts (named *EF1* and *EF2*) against a *Fusarium proliferatum* (AACC0215) strain that causes diseases to many economically important plants and synthesizing diverse mycotoxins. In this work, we aimed to identify the most appropriate concentration between the tested two olive leaf extracts to develop a safe, stable and efficient drug delivery system. Qualitative and quantitative analyses of the two olive leaf extracts by (HPLC) were performed. Furthermore, we also evaluated the antifungal effects of the two leaf extracts when encapsulated in chitosan-tripolyphosphate nanoparticles. The major compound in both *EF1* and *EF2* was oleuropein, with 336 and 603 mg/g, respectively, however, high concentrations of flavonoid were also present. *EF1* and *EF2* showed a concentration depended effect on *F. proliferatum* (AACC0215) viability. Our results showed a great efficacy of *EF1*/nanoparticles at the higher concentration tested (12X) against the target species. In this case, we observed an inhibition rate to both germination and growth of 87.96 and 58.13%, respectively. We suggest that *EF1* olive leaf extracts, as free or encapsulated in chitosan-tripolyphosphate nanoparticles, could be used as fungicides to control plant diseases. Finally, future application of these findings may allow to reduce the dosage of fungicides potentially harmful to human health.

## Introduction

Nanoparticle formulation is beneficial in different fields including electronics, textiles, mobile phones, food, paper, robotics, fertilizers, pesticides, and agrochemical industries. In recent years, an increased interest has been developed for natural polymers which have a versatility due to their chemical, physical and functional properties. The wide range of potential applications has led to their use in various fields of research, mainly in the biomedical, cosmetics, food and pharmaceuticals (Agnihotri et al., 2004; Manna and Patil, 2009).

Chitosan (CS) has emerged as one of the most promising polymers for the formation of nanoparticles (NPs) (Kashyap et al., 2015), mainly due to its biodegradable and biocompatible properties, its moderate or lack of toxicity to animals and humans, and for its antimicrobial and antifungal activity (López-León et al., 2005; Zhou and Chen, 2008; Akamatsu et al., 2010). Chitosan nanoparticles have gained considerable popularity as a carrier for the active ingredient delivery for various applications owing to their biocompatibility, biodegradability, high permeability, cost-effectiveness, and non-toxicity (Shukla et al., 2013). Various procedures can be employed to synthesize CSNPs, such as emulsion formation, coacervation, spray drying, ionotropic gelation. The method selected is mainly dependent on the substances encapsulated, and the route of administration. So, by varying the concentration and the molecular weight of the polymer and by using copolymers and crosslinking agents, efficient delivery systems for the pharmaceutical, biomedical and agricultural industry could be obtained (Höhne et al., 2007; Nasti et al., 2009).

In agriculture, NPs could be used as vectors to control release of agrochemicals, such as fertilizers, pesticides, herbicides and plant growth regulators (Cota-Arriola et al., 2013). Plants are continuously exposed to a series of pathogenic microorganisms such as fungi, oomycetes and bacteria, which can attack the plant both above and below ground (Buhtz et al., 2015) and cause the evolution of devastating epidemics and significant yield losses of annual crops, seriously affecting the economy. Several fungal species belonging to the genus *Fusarium* are known for their ability to colonize a wide variety of host plants, such as tomatoes, potatoes, cereal and tobacco (Desjardins, 2003; Schweigkofler et al., 2004; Alves-Santos et al., 2007; Nguyen et al., 2016). The most common symptoms of the disease are wilting, yellow leaves, dry collar, chlorosis, premature leaf drop, browning of the vascular system and growth arrest. When the disease spreads to the whole plant, necrosis and death occurs (Trapero-Casas and Jiménes-Dìaz, 1985). *Fusarium* produces mycotoxins which can have an important role in pathogen virulence during infection of the plant (Nguyen et al., 2016). The control of these fungi, responsible for pre- and post-harvested diseases of agricultural products, is an issue that remains unresolved, along with the excessive environmental impacts of chemicals to tackle this problem. Current efforts are focused to search new strategies and effective alternatives for microbial control and to reduce the excessive use of synthetic fungicides which negatively impact the environment and human and animal health (Cota-Arriola et al., 2013; Rodriguez-Maturino et al., 2015).

Plants have been a rich source of bioactive compounds for millennia, while the use of plant derivatives to produce nanobiotechnological formulations has gained scientific and technological importance in recent years (Joanitti and Silva, 2014). *Olea europaea* belongs to the Oleaceae family and it is native of the Mediterranean region. Olive oil, fruit and leaves have been recognized as important components of medicine and of a healthy diet. The extract from olive leaves were reported to have anticancer, antioxidative and anti-inflammatory properties (Le Toutour and Guedon, 1992; Anter et al., 2011). In addition to the health benefits described above, it is claimed that extracts from olive leaves may aid in the treatment of a broad range of infectious diseases. They have important pharmacological properties attributable primarily to the phenolic content (Omar, 2010). The main phenolic compound present in the leaves and fruits of olive tree is oleuropein (*Ole*) (Bianco et al., 1999; Goldsmith et al., 2015) and the detectable amount ranges from 17% to 23%, depending on the harvesting period (Le Toutour and Guedon, 1992). Olive leaves extract is characterized by a high content of phenolic compounds and flavonoids such as *Ole*, hydroxytyrosol and their derivatives (Zorić et al., 2016) and luteolin 7-glucoside and their derivatives (Sudjana et al., 2009). The antimicrobial activity of *Ole* and leaf extracts has been examined previously (Markin et al., 2003; Sudjana et al., 2009).

In this work, we aimed to identify the most appropriate concentration between the tested two olive leaf extracts to develop a safe, stable and efficient drug delivery system. These CSNPs were synthesized by a chemical route and displayed certain characteristics defined by preparation conditions. The physical and chemical characterization of the nanoformulation such as mean particle size, zeta potential values, polydispersity index (PDI), and EE were evaluated. In this study, the CSNPs antifungal effect was evaluated against *F. proliferatum* (AACC0215) strain through an *in vitro* assay, looking at different concentrations and preparations of the olive leaf extracts.

## Experimental

### Sample Preparation

The plant material used for the extraction is represented by fresh leaves of Carolea cultivar collected in November 2015 from plants grown in the Botanical Gardens of the University of Calabria, Arcavacata di Rende (CS) (GPS coordinates: latitude 39.357548; longitude 16.228990). The plants were identified by Dr. Nicodemo Passalacqua curator of the Botanical Gardens of University of Calabria.

### Extraction and Characterization of the Olive Leaf Extracts

The method used for the extraction of olive leaf extracts is that described in Muzzalupo et al. (2011) with some modifications. Olive leaves (20 grams corresponding to about 100 leaves) were homogenized in 100 mL of a mixture of acetone and methanol in a ratio of 1:1 (v/v). The homogenization was carried out using an Ultra-TURRAX^®^ (IKA, Seneco Science, Milan, Italy) for 5 min at room temperature. The homogenized mixture was vacuum filtered, and the liquid portion was recovered. The pellet was re-homogenized with the previous mixture and the process was repeated a further two times. The filtrate obtained was evaporated to dryness with a rotavapor (Strike 202 Rotary Evaporator, Steroglass, Perugia, Italy) and resuspended with 80 mL of distilled water. The filtrate was washed, in the separating funnel, with different solvents with increasing polarity: *n*-hexane, ethyl ether, chloroform and ethyl acetate. All solvents used are pure (ACS grade solvents, Sigma-Aldrich, Milano, Italy). The washings with *n*-hexane and ethyl ether were discarded, instead those from the chloroform and ethyl acetate phases were recovered and kept separate.

The extract obtained from the chloroform was referred to as “*leaf extract 1” (EF1)*, while that derived from ethyl acetate as “*leaf extract 2” (EF2)*. The two extracts were made anhydrous with sodium sulfate, filtered and evaporated to dryness and stored at −20°C in the dark.

#### Determination of Total Phenolic Compounds

The total phenolic content of each extract was determined spectrophotometrically at 750 nm using Folin-Ciocalteu reagent (Fuentes et al., 2012). To 1 mL of the sample to be tested were added 0.5 mL of Folin-Ciocolteu (Sigma-Aldrich) and left in the dark for 5 min. Subsequently 3 mL of Na_2_CO_3_ (Sigma-Aldrich) at 20% and 5.5 mL of distilled water were added. After 20 min, spent in the dark and at room temperature, samples were centrifuged at 3,500 rpm for 10 min. A calibration curve was calculated using pure *Ole* (Extrasynthèse, ZI Lyon-Nord, Genay, France). The total phenolic compounds are expressed as *Ole* milligrams per grams of extract.

#### Identification of Phenolic Compounds Contained in Each Extract by HPLC

Both extracts (*EF1* and *EF2*), solubilized in methanol, were characterized performing HPLC analysis. The procedure used is that reported by Montedoro et al. (1992): HPLC JASCO LC-2000 plus equipped with a pump PU-2080 and UV-2075 detector (JASCO), with a RP-18 column Spherisorb ODS-2 (160 mm x 4.6 mm, Waters, Vimodrone, Italy) and injection volume of 20 μL; the flow rate was 1 mL/min at room temperature; the mobile phase used was 2% acetic acid in water (A) and methanol (B) for a total running time of 45 min, and the gradient conditions were as follows: 95% A-5% B for 2 min, 75% A-25% B for 8 min, 60% A-40% B for 10 min, 60% A-50% B for 10 min and 0% A-100% B for 10 min, until it stops; the eluents were detected at 280 nm. As phenolic standards were used: *Ole*, verbascoside, luteolin-4′-O-glucoside, luteoloside, luteolin, apigenin-7-O-glucoside and apigenin, all purchased from Extrasynthése.

### Preparation and Characterization of the CSNPs

The CSNPs were prepared by ionotropic gelation method, reported by Rampino et al. (2013), with some modifications. Dispersions of chitosan were prepared, at a concentration of 1 mg/mL, by dissolving the medium molecular weight chitosan (50,000–190,000 Da, 75–85% deacetylated, Sigma Aldrich) in a solution of hydrochloric acid to 0.04% (v/v) and then stirring for 1 h. The pH of the CS solution was adjusted to 5.5 by NaOH. 1 mL of *Ole* or *EF1* or *EF2* water solution was added to 5 mL of the chitosan solution leaving it under stirring for a few minutes and adjusting the pH to 5.5. Tripolyphosphate (Sigma Aldrich) was dissolved in distilled water to a final concentration of 2 mg/mL and was added dropwise to the chitosan solution in a volumetric ratio of 1:5. The resulting solution was stirred for 30 min at room temperature. Moreover, CSNPs without the leaf extract were prepared. All formulations assayed (1X, solutions and carried CSNPs) were prepared at a final concentration of 100 mg/L of *Ole* (Table 1).

**TABLE 1:** Phenolic compounds (Oleuropein – *Ole*; leaf extract 1 – *EF1*; leaf extract 2 – *EF2*) used as free or encapsulated in CSNPs, for *in vitro* assays against *Fusarium proliferatum* (AACC0215).

**Code samples**	**Composition**	**Concentration tested (1X) [mg/L]**
*Ole*	Oleuropein (standard)	100
*EF1*	Oleuropein	100.0^a^ ± 1.0
	Luteolin-4-*O*-glucoside	18.0^a^ ± 0.1
	Luteolin-7-glucoside	43.0^a^ ± 0.2
	Verbascoside	5.0^a^ ± 0.1
	Phenols unidentified	137.0^a^ ± 1.2
	***Totals phenols***	**303.0^a^ ± 2.6**
*EF2*	Oleuropein	100.0^a^ ± 1.0
	Luteolin-4-*O*-glucoside	14.0^a^ ± 0.1
	Luteolin-7-glucoside	25.0^b^ ± 0.2
	Verbascoside	8.0^a^ ± 0.1
	Phenols unidentified	35.0^b^ ± 0.2
	***Totals phenols***	**182.0^b^ ± 1.6**

*Means with different letters for the same quality parameter differ significantly by Tukey’s test (*p* < 0.05). Data are average ±SEM (*n* = 3).*

All nanoformulations were characterized in terms of particle size, size distribution, PDI and zeta potential using a Zetasizer ZS (Malvern Instrument Ltd., Malvern), based on the DLS technique. DLS measurements of the samples were performed at 25°C with a detection angle of 90°.

#### Evaluation of Drug Loaded Efficiency

The extracts UV spectrum had a single maximum of absorption at 280 nm and this aspect allowed us to treat the extracts as a single component. The EE was calculated using the following formula (Shi et al., 2014):

$$E\,E\%=\frac{t\,o\,t\,a\,l\,a\,m\,o\,u\,n\,t\,o\,f\,d\,r\,u\,g-f\,r\,e\,e\,d\,r\,u\,g}{t\,o\,t\,a\,l\,a\,m\,o\,u\,n\,t\,o\,f\,d\,r\,u\,g}\times 100$$

Where drug means the phenolic compounds under study (*EF1*, *EF2* and *Ole*). Each preparation was filtered using the syringe filters with a porosity equal to 0.2 μm (Millipore, Italy). 100 μL of filtrate are taken and brought to a final volume of 5 mL with distilled water. The amount of free and total drug was calculated by using the V-530 spectrophotometer (JASCO) at 280 nm (Mazzotta et al., 2020).

#### *In vitro* Olive Leaf Extracts Release

The release of *Ole* and leaf extracts from CSNPs was estimated using the method reported in Varuna Kumara and Basavaraj (2015), with some modifications. 2 mL of CSNPs/*Ole* or CSNPs/*EF2* were taking and placing in pre-treated dialysis tubes Spectra/Por 4 (MWCO: 12–14 kD, Spectrum Laboratories, Inc., Canada). These were dipped into 50 mL of PBS solution (pH = 5.9) and left to stir at room temperature. At predetermining time points, 2 mL of the medium were taken and replaced with the same volumetric amount of fresh PBS. The solution was analyzed by UV-VIS spectrophotometry to evaluate the drug content.

### Assessment of Antifungal Activity

#### Used Strains

In order to evaluate the antifungal activity of prepared extracts, the *F. proliferatum* (AACC0215) strain were used. Isolation and identification of the strain was described in a previous study (Muto et al., 2014). In brief, *F. proliferatum* (AACC0215) was isolated from colonized cloves of garlic (*Allium sativum*) collected at Altomonte, Cosenza, Italy in the year 2013, and taxonomically characterized as described in Muto et al. (2014). This strain was sub-cultured on potato dextrose broth (PDB) and incubated in darkness at 24°C. The suspension was diluted to a concentration of 1 × 10^5^ spores/mL. Afterward it was divided into 1.5 mL aliquots and stored at −80°C in 25% glycerol (Steinkellner and Mammerler, 2007).

#### *In vitro* Test for the Evaluation of Germination

**FIGURE 1:**
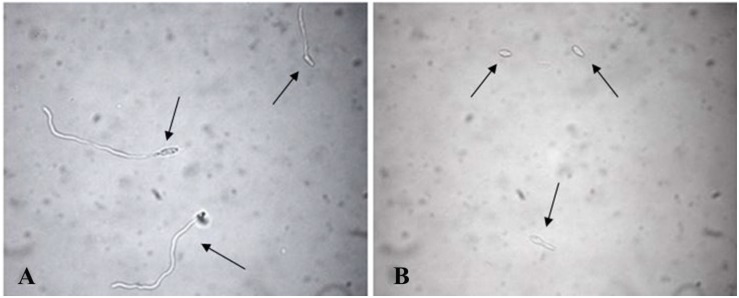
Conidia of *Fusarium proliferatum* (AACC0215) germinated in distilled water **(A)** and in presence of *Ole* **(B)** after 24 h. The samples were observed under an optical microscope at X400 magnification. The “arrow heads” indicate the germinated **(A)** and the non-germinated conidia **(B)**.

To verify the ability and success of *F. proliferatum* (AACC0215) to germinate in the presence of *EF1*, *EF2*, and *Ole*, different tests were performed using multiwell plates for cell cultures (Sigma-Aldrich). Inside of each well 200 μL of test preparation and 10 μL of conidial suspension at a concentration of 1 × 10^5^ conidia/mL were added. Table 1 shows the composition of the analyzed individual preparations, respectively, containing *Ole*, *EF1* and *EF2* in solution or carrier to the CSNPs. The concentration used for each treatment was 3, 6, 9, and 12 times the initial one. As a control, the fungus was inoculated into the wells containing sterile water. The *F. proliferatum* (AACC0215) was incubated at 25–27°C in the dark and under aerobic conditions. After 24 h, 20 μL of each solution were taken to prepare slides by using a Malassez cell. The samples were observed under an optical microscope (DMRB Leica Microsystems, Milan, Italy) at × 400 magnification, equipped with a digital camera (DFC490 Leica Microsystems). The evaluation of germinated conidia and index of germination were performed according to Benslim et al., 2016. The conidia were considered as germinated when the germ tube length exceeded the diameter of the conidium (Figure 1; Boch et al., 1999; Rosengaus et al., 2000).

For each slide, a total of 500 conidia were counted, by determining the percentage of inhibition rate (*% IRg*) by using the following formula:

%⁢I⁢R⁢g=Ng∘erminatedconidiaincontrol-N∘g⁢e⁢r⁢m⁢i⁢n⁢a⁢t⁢e⁢d⁢c⁢o⁢n⁢i⁢d⁢i⁢a⁢i⁢n⁢t⁢r⁢e⁢a⁢t⁢m⁢e⁢n⁢t⁢sN⁢g∘⁢e⁢r⁢m⁢i⁢n⁢a⁢t⁢e⁢d⁢c⁢o⁢n⁢i⁢d⁢i⁢a⁢i⁢n⁢c⁢o⁢n⁢t⁢r⁢o⁢l×100

#### *In vitro* Inhibition to Growth

The essays were conducted as described in Taskeen-Un-Nisa et al. (2011) with small modifications. Assays were performed in Petri plates containing 25 mL of PDA supplemented with streptomycin and ampicillin, at a final concentration of 6 mg/L each. At the center of the plates, a sterile polycarbonate filter with 0.8 μM porosity (Isopore Membrane Filters, Millipore) was placed on the PDA and 50 μL of the solutions to be tested were added on it.

In order to test the *in vitro* activity of the individual preparations at increasing concentrations, volumes of 3, 6, 9, and 12 times more than the starting solution described in Table 1 were loaded on the polycarbonate filter in the Petri capsule. In this way a thin and uniform film was formed on the surface.

PDA plates, to which a filter of 50 μL of ethanol had been added, were used as controls. Subsequently, the dry filters were inoculated at the center with 4 μL of conidial suspension at a concentration of 1 × 10^5^ spores/mL and they were incubated in the dark at 24°C under aerobic conditions for 6 days. At the end of incubation, the capsule was photographed, and the image was analyzed by using the ImageJ software (vers. 1.49v National Institutes of Health, United States) to calculate the area, expressed in square millimeters, occupied by the mycelium.

The percentage of growth inhibition *(I%)* was calculated using the following formula (Chin Ming et al., 2015):

$$I\%=\frac{A_{C}-A_{T}}{A_{C}}\times 100$$

Where *A*_C_ represents the average value of the area of mycelium used as a control and *A*_T_ the average area value of the mycelium inoculated on plates treated with the individual preparations (*Ole*, *EF1* and *EF2* free and carrier to the CSNPs) (Kaiser et al., 2005; Taskeen-Un-Nisa et al., 2011).

### Statistical Analysis

Statistical analysis was performed with XLSTAT v.2016. All data obtained from *in vitro* tests were compared by using One-way ANOVA, with Tukey’s multiple comparison test. All results are the mean of at least three individual experiments. All the values obtained from chemical analysis and biological tests are calculated from triplicate data were expressed as means ±standard error.

## Results and Discussion

### Analysis of Olive Leaf Extracts

**FIGURE 2:**
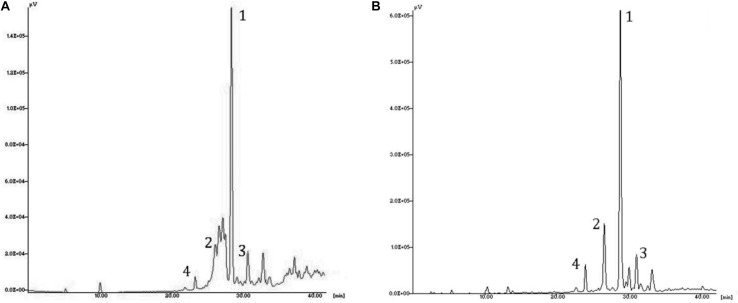
HPLC chromatogram of the leaf extracts *EF1* **(A)** and *EF2* **(B)**. Assignment of the compounds to the identified peaks: (1) oleuropein, (2) luteolin-7-glucoside, (3) luteolin-4′-O-glucoside; (4) verbascoside.

The identification of the phenolic compounds was carried out by comparing the retention times obtained from the HPLC analysis of the olive leaf extracts and those of the available standards. The results of the HLPC analysis of *EF1* and *EF2* showed a different content of phenols, in relation to the extraction procedure followed and their hydrophilicity (Table 2). In chloroform (*EF1*) and ethyl acetate (*EF2*) olive leaf extracts four phenol compounds were identified and quantified: *Ole*, verbascoside, luteolin-4′-O-glucoside, and luteolin-7-glucoside (Figure 2). Phenol compounds apigenin, luteolin, and apigenin-7-O-glucoside were not detected.

**TABLE 2:** Quantitative and qualitative analysis of *EF1* and *EF2* phenolic compounds analyzed by HPLC.

**Compound**	**N° peak**	**T_R_ (min)**	**Yield in *EF1* (mg/g)**	**Yield in *EF2* (mg/g)**
Verbascoside	**4**	23.3	18.5^a^ ± 3.3	47.9^b^ ± 4.7
Luteolin-7-glucoside	**2**	26.6	143.5^a^ ± 12.3	147.8^a^ ± 13.4
Oleuropein	**1**	28.3	335.7^a^ ± 22.3	603.2^b^ ± 42.3
Luteolin-4-*O*-glucoside	**3**	30.9	61.4^a^ ± 5.3	82.7^a^ ± 7.5
Unidentified*	–	–	460.3^a^ ± 32.3	118.4^b^ ± 10.2

Means with different letters for the same quality parameter differ significantly by Tukey’s test (*p* < 0.05). Data are average ±SEM (*n* = 3). *Sum of (over 30) unidentified substances.

The most abundant compound of chloroform and ethyl acetate olive leaf extracts is *Ole*, with 336 mg/g and 603 mg/g of extract, respectively. Furthermore, in both olive leaf extracts, there are numerous (over 30) unidentified phenolic compounds, which represented 460 and 118 mg/g of extract, respectively (Table 2).

The *EF2* extract had a lower level of total phenols when compared to *EF1* extract, 0.75 g and 1.04 g of extract, respectively. This suggests that most of the compounds present in the olive leaves were less hydrophilic phenols (Tan et al., 2014). Consistent with this, chloroform is less polar than ethyl acetate. The obtained results are in line with previously published data for *O. europaea* leaves, where *Ole* was identified as the major phenol compound extract (Yateem et al., 2014).

### Nanoparticles Preparation and Characterization

Chitosan nanoparticles were prepared by ionotropic gelation with the dropwise addition of tripolyphosphate to a chitosan solution. Formation of NPs occurs quickly upon mixing tripolyphosphate and chitosan solutions and this is due to the electrostatic interactions between the positively charged primary amino groups of chitosan and the negatively charged groups of tripolyphosphate (Servat-Medina et al., 2015). The different formulations of CSNPs, containing different concentrations of *Ole*, showed good stability over time. They were stored in the dark at 4°C. When monitored after 30–40 days, they did not show sedimentation, creaming or flocculation. The particle size, PDI, zeta potential and EE are displayed in Table 3 for all nanoformulations.

**TABLE 3:** The particle size, polydispersity index (PDI), zeta potential (Z-P), and encapsulation efficiency (EE%).

**Formulation**	**Particle size (nm)**	**PDI**	**Z-P (mV)**	**EE (%)**
CSNPs	260.3^a^ ± 29.4	0.257^a^ ± 0.031	25.0^a^ ± 1.72	–
CSNPs/Ole	254.6^a^ ± 20.7	0.250^a^ ± 0.025	16.9^b^ ± 0.71	62.2^a^ ± 3.4
CSNPs/EF1	258.7^a^ ± 17.2	0.207^a^ ± 0.042	15.7^b^ ± 1.31	94.5^b^ ± 5.2
CSNPs/EF2	269.4^a^ ± 36.3	0.229^a^ ± 0.032	11.5^b^ ± 2.08	73.1^c^ ± 4.7

*Means with different letters for the same quality parameter differ significantly by Tukey’s test (*p* < 0.05). Data are average ±SEM (*n* = 3).*

The NPs have always shown dimensions between 250 to 270 nm. As for the PDI values, these are always lower than 0.3 and this indicates a clear homogeneity of the system. No significant differences were seen in the Z-potential except for CSNPs/*EF2* whose values are lower than those of CSNPs, indicating a greater presence of negative charge density. This result could possibly be related to the chemical nature of the unidentified compounds present in different percentages in the two leaf extracts.

During the formation of NPs, bioactive molecules are trapped both inside and on the surface of such particles. However, there is an initial burst release probably due to the drug on the surface, followed by a prolonged release (Bahreini et al., 2014).

**FIGURE 3:**
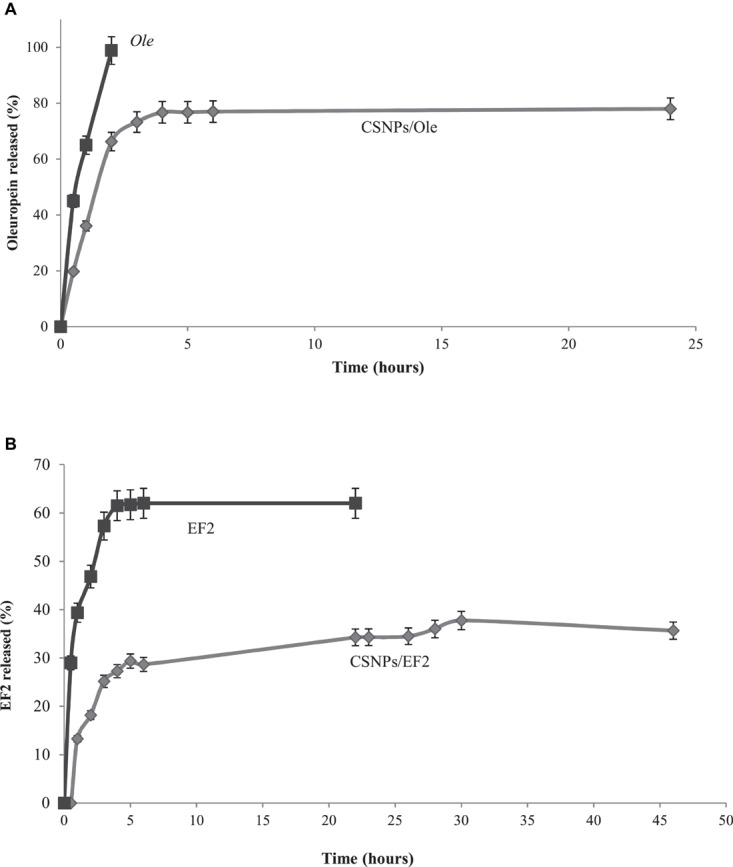
**(A)** *In vitro* release profile of the commercial oleuropein in solution (*Ole*) (■) and in chitosan nanoparticles (CSNPs/*Ole*) (•). Data are average ±S.E.M. (*n* = 3). **(B)** *In vitro* release profile of EF2 leaf extract in solution (■) and in chitosan nanoparticles (CSNPs/EF2) (•). Data are average ± SEM (*n* = 3).

Commercial *Ole* is completely released after 2 h of dialysis, while *Ole* in CSNPs is released more slowly, reaching the maximum value of 77% after 6 h (Figure 3A). *EF1* after 45 h of dialysis against water has a negligible release, probably due to a lower hydrophilia. *EF2* extract, both in solution and in NPs, is released in a lower percentage and more slowly than *Ole* (Figure 3B). Specifically, the free solution *EF2* reaches a release value equal to 62% after 5 h, instead the release values of *EF2* encapsulated in the NPs, reach the maximum value, equal to 38%, only after 30 h. This behavior may, probably, depend on the presence of lipophilic compounds in leaves, including lipophilic phenols, that are not allowed to pass into PBS.

### Assessment of Antifungal Activity

#### *In vitro* Test for the Evaluation of Germination

The antifungal activity of *Ole*, *EF1*, and *EF2* in solution or carrier on the CSNPs on conidial germination of *F. proliferatum* (AACC0215) is shown in Table 4. The obtained results were compared with distilled water. All formulations show an inhibition percentage of conidial germination versus control.

**TABLE 4:** *In vitro Fusarium proliferatum* (AACC0215) percentage of inhibition rate (% IRg) in the presence of pure chitosan nanoparticles (CSNPs), oleuropein (*Ole*), leaf extract 1 (*EF1*) and leaf extract 2 (*EF2*) free in solution or encapsulated into NPs.

	**IRg (%)**				

***Formulation***	***1X***	***3X***	***6X***	***9X***	***12X***
CSNPs	48.71*^a^*±0.49	48.71*^a^*±0.49	48.71*^a^*±0.49	48.71*^a^*±0.49	48.71*^a^*±0.49
*Ole*	10.19*^a,b^*±4.01	12.96*^b,c^*±1.26	22.11*^a,b^*±4.26	47.19*^c,d^*±2.62	58.50*^d,e^*±1.58
CSNPs/*Ole*	13.23*^a,b^*±1.34	28.00*^b,c^*±1.01	66.28*^c,d^*±2.04	67.41*^c,d^*±2.28	62.75*^c,d^*±4.07
EF1	68.72*^a,b^*±1.72	68.48*^a,b^*±2.40	84.87*^b,c^*±2.03	86.18*^b,c^*±1.38	84.66*^b,c^*±2.80
CSNPs/*EF1*	52.45*^a,a^*±3.16	55.01*^a,b^*±2.74	57.42*^a,b^*±2.90	61.17*^b,b^*±4.19	87.96*^c,c^*±2.50
EF2	44.10*^a,a^*±4.56	50.37*^b,b^*±1.03	58.90*^c,c^*±4.33	26.55*^d,d^*±2.66	36.27*^e,e^*±3.51
CSNPs/*EF2*	37.00*^a,b^*±4.19	54.45*^b,c^*±3.11	62.39*^c,d^*±1.68	57.37*^d,e^*±2.07	67.81*^e,d^*±1.11

*The germination tests were conducted in sterile water. The first letter refers to the significance of the same preparation for the different concentrations, the second is related to the significance of all the preparations for the same concentration. Means with different letters for the same quality parameter differ significantly by Tukey’s test (*p* < 0.05). Data are average ±SEM (*n* = 3).*

The maximum inhibition percentage of conidial germination was obtained at the 12X concentration (87.96%), in CSNPs/*EF1* formulation, while the lowest one was obtained at the 1X concentration (10.19%) of *Ole* solutions. The empty CSNPs markedly reduced conidial germination of *Fusarium* compared to the control, equal to 48.71%. The mechanism of action could be based on the electrostatic interaction between the amine group of chitosan and the negatively charged compounds (phospholipids, proteins, amino acids) of the cell membrane of fungi (Rabea et al., 2003; Liu et al., 2004).

The results obtained have shown a reduction in the germination capacity of the conidia, depending on the concentrations employed, except for the solution prepared with *EF2* (Table 4). A reduction in the germination capacity of conidia was recorded as the concentration increased. In fact, in the presence of *Ole* the highest percentage of inhibition of germination was obtained at the highest concentration used 12 mg/L (12X) with a percentage of 58.50%, while with the CSNPs/*Ole* complex the maximum (67.41%) was obtained at the concentration of 900 mg/L (9X).

Regarding the leaf extract *EF1*, the germination capacity of the conidia is very limited compared to *Ole*, reaching a maximum value of 68.72% at the lowest concentration (1X). However, in the CSNPs/*EF1* system, the inhibition of germination increases to between 52.45% (1X) and 61.17% (9X) (Table 4). The formulation that records the greatest reduction in germination capacity of *F. proliferatum* (AACC0215) conidia, reaching 87.96% (12X), is CSNPs/*EF1* at the highest concentration assayed. Considering the solution containing only CSNPs, it produced an *IRg%* equal to 48.71. This result is the same in all treatments because there is no drug inside the CSNPs, so the concentration of each component of the NPs is the same in all the concentrations tested.

The CSNPs/*Ole* and CSNPs/*EF1* complex results suggest that these systems act with an enhanced effect at the highest concentrations. It is the behavior of the CSNP/*Ole* formulations obtained with a low *Ole* content (1X and 3X) that have not shown the expected performance with respected to IRg%. The IRg% decreases significantly compared to the control, this result can be attributed to a decrease of the CSNPs chelated positive charges by the hydroxyl groups of the *Ole*. With respect to *EF2*, the obtained results showed a decrease in the inhibition of germination at the highest concentrations tested with a value of 26.55% at the concentration 9X. The increased germination capacity may depend on the presence of impurity within the extract that is likely to be used as a source of energy from the fungus and which could stimulate germination of spores.

#### *In vitro* Inhibition to Growth

The results obtained have shown an antifungal activity for almost all the analyzed samples, with the exception of six formulations (*CSNPs, Ole* 1X, and 3X; CSNPs*/OLE* 1x; CSNPs*/EF1* 1x; *EF2* 9x, Table 5) that induce a stimulation in growth.

**TABLE 5:** *In vitro Fusarium proliferatum* (AACC0215) percentage of growth inhibition (% I) in the presence of pure chitosan nanoparticles (CSNPs), oleuropein (*Ole*), leaf extract 1 (*EF1*) and leaf extract 2 (*EF2*) free in solution or encapsulated into NPs.

	**I (%)**				

***Formulation***	***1X***	***3X***	***6X***	***9X***	***12X***
CSNPs	−22.41*^a,a^*±5.43	17.62*^b,a^*±0.48	21.57*^b,a^*±7.31	40.85*^c,a^*±1.34	41.93*^c,a^*±3.17
*Ole*	−27.40*^a,a^*±10.15	−13.41*^a,b^*±16.43	58.02*^b,b^*±1.91	52.43*^b,b^*±4.78	33.45*^c,a^*±5.88
CSNPs/*Ole*	−27.96*^a,a^*±2.42	22.06*^b,c^*±7.91	66.31*^c,b^*±10.47	64.99*^c,c^*±1.87	51.62*^c,b^*±7.30
EF1	49.38*^a,b^*±11.41	31.48*^b,b^*±1.67	46.56*^a,b^*±0.29	53.34*^a,b^*±4.99	48.52*^a,b^*±2.88
CSNPs/*EF1*	−7.38*^a,a^*±5.66	10.38*^b,c^*±1.73	28.45*^c,a^*±3.79	44.37*^d,b^*±2.57	58.13*^e,c^*±3.05
EF2	4.64*^a,b^*±0.93	6.99 ± 5.08 *a,b*	23.53*^b,a^*±4.98	−16.41*^c,b^*±5.77	3.55*^a,b^*±0.67
CSNPs/*EF2*	9.85*^a,c^*±3.13	15.02*^a,c^*±1.31	32.60*^b,b^*±1.73	19.98*^c,c^*±5.13	32.20*^b,c^*±1.96

*The first letter refers to the significance of the same preparation for the different concentrations, the second is related to the significance of all the preparations for the same concentration. Means with different letters for the same quality parameter differ significantly by Tukey’s test (*p* < 0.05). Data are average ± SEM (*n* = 3).*

In almost all the concentrations tested, the empty CSNPs inhibited *F. proliferatum* (AACC0215) growth by 17.62, 21.57, 40.85, and 41.93%, respectively. Pure *Ole* and *EF1* at higher concentrations, both in solution and in CSNPs, exhibit a higher inhibition rate of growth than *EF2* (Table 5). *Ole* showed good antifungal activity at concentrations of 600 mg/L and 900 mg/L (6X and 9X) with a *I%* equal, respectively, at 58.02% and 52.43% when administered directly and at 66.31 and 64.99% if encapsulated in CSNPs. *EF1* fungicidal effect was quite similar to all concentrations used. *EF1* solution has shown inhibitory activity already at the lowest concentration (1X) with a *I%* equal to 49.38%. When *EF1* was combined with CSNPs, *F. proliferatum* (AACC0215) growth inhibition was found to be directly proportional to the concentration used, reaching a value of 58.13% at the highest concentration (12X). *EF2* pure exhibited lower antifungal activity with a maximum inhibition value of 23.53% at concentration 6X. However, the maximum of growth inhibition percentage was obtained at 6X and 12x concentration by using the complex CSNPs/*EF2* (32.60 and 32.20%, respectively).

All formulations containing CSNPs have an activity of *I%* greater or not significant compared to the control. Only in the case of CSNPs/*EF2* the higher concentrations appear to have significantly lower values than the control. These results, as mentioned above, is probably due to the presence of impurities of the extract *EF2* that the mushroom uses as a source of energy and which stimulate its growth.

In this study, it was observed that the tested phenolic compounds exert a cytotoxic activity *in vitro* against *F. proliferatum* (AACC0215) and this activity increase when they are complexed with CSNPs. These results are in line with other studies in which phenols extract from olive leaves show an antifungal activity (Markin et al., 2003; Steinkellner and Mammerler, 2007; Goldsmith et al., 2015; Rodriguez-Maturino et al., 2015; Zorić et al., 2016). As reported by Ansari et al. (2013) the phenols antimicrobial activity could be due to a synergistic action of the antioxidant and chelating power of the hydroxyl groups of the phenolic ring that form hydrogen bonds with cell wall proteins of microorganisms. Chitosan also exhibits antimicrobial activity based on the electrostatic interaction between the amine group of chitosan and the negatively charged compounds (phospholipids, proteins, amino acids) of the cell membrane of fungi (Rabea et al., 2003; Liu et al., 2004). Therefore, the interaction between phenolic compounds complexed with CSNPs cause an alteration of the integrity and permeability of the microbial cell.

## Conclusion

Olive leaf extract-encapsulated CSNPs were obtained by ionotropic gelation method. The characterization of synthesized NPs showed that the size of leaf extract/CSNPs was 254.6–269.4 nm, the EE ranged from 62.2 at 94.5% and Zeta potential varied from 11.5 at 25.0 mV. As for the polydispersity index values, the lower was 0.207 and this indicates a clear homogeneity of the system. The nanoformulation thus achieved may be explored for the target delivery of phenols for disease control.

Considering the highest concentration (12X) tested, leaf extract/CSNPs showed greater efficacy than pure extracts (*EF1* and *EF2*) and the commercial formulation (*Ole*) against *F. proliferatum* (AACC0215). We suggest that the *EF1* olive leaf extracts, as free or encapsulated in chitosan-tripolyphosphate nanoparticles, could be used as fungicides to control plant diseases. Finally, future application of these findings may allow to reduce the dosage of fungicides potentially harmful to human health.

## Data Availability Statement

All datasets generated for this study are included in the article/supplementary material.

## Author Contributions

IM, AC, NP, and RM designed the research, analyzed the data, and discussed the results. GB performed the research and discussed the results. All authors contributed to improving the manuscript and approved the final manuscript.

## Conflict of Interest

The authors declare that the research was conducted in the absence of any commercial or financial relationships that could be construed as a potential conflict of interest.

## Abbreviations

CS: chitosan
CSNPs: chitosan nanoparticles
DLS: dinamic light scattering
EE: encapsulation efficiency
EF1: leaf extract 1
EF2: leaf extract 2
HPLC: high performance liquid chromatography
NPs: nanoparticles
Ole: oleuropein
PBS: phosphate-buffered saline
PDA: potato dextrose agar
PDI: polydispersityindex.
